# Peripheral blood CD4^pos^CD25^pos^FoxP3^pos^ cells and inflammatory cytokines as biomarkers of response in rheumatoid arthritis patients treated with CTLA4-Ig

**DOI:** 10.1186/s13075-022-02827-5

**Published:** 2022-06-15

**Authors:** Gremese Elisa, Barbara Tolusso, Luca Petricca, Clara Di Mario, Maria Rita Gigante, Gianfranco Ferraccioli, Stefano Alivernini

**Affiliations:** 1 Division of Clinical Immunology, Catholic University of the Sacred Heart, Fondazione Policlinico Universitario A. Gemelli IRCCS, Via Giuseppe Moscati 31, 00168 Rome, Italy; 2 Immunology Core Facility, Gemelli Science Technological Park, GSTeP, Fondazione Policlinico Universitario A. Gemelli IRCCS, Rome, Italy; 3 Università Cattolica del Sacro Cuore, Rome, Italy; 4 Division of Rheumatology, Catholic University of the Sacred Heart, Fondazione Policlinico Universitario A. Gemelli IRCCS, Largo Agostino Gemelli 1, 00168 Rome, Italy

**Keywords:** Rheumatoid arthritis, Biomarkers, CTLA4-Ig

## Abstract

**Background:**

Prognostic biomarkers of treatment response to distinct biologic disease-modifying anti-rheumatic drugs (b-DMARDs) are still lacking within the management of rheumatoid arthritis (RA).

**Methods:**

Thirty-four b-DMARDs naive RA patients, divided by disease duration into early (cohort 1) and long standing (cohort 2), received CTLA4-Ig. At study entry, and every 3 months for 1 year, each patient underwent peripheral blood (PB)-derived CD4^pos^ cell subpopulation assessment by flow cytometry, STAT3 and STAT5 expression by RT-PCR and IL-6, IL-12p70, TGFβ, and IL-10 serum levels by ELISA. The DAS and CDAI remission was assessed at 6 and 12 months.

**Results:**

DAS- and CDAI-defined remission within 12 months was achieved by 16 (47.1%) and 8 (23.5%) RA patients, respectively. Considering the whole RA cohort, CTLA4-Ig induced a significant decrease of IL-6 serum levels from baseline to 6 and 12 months, as well as of PB CD4^pos^CD25^pos^FoxP3^pos^ cells at 6 and 12 months, and of CD4^pos^IL17^pos^ cells after 12 months. PB CD4^pos^ cells of RA patients showed higher STAT3 and STAT5 expression than healthy controls, which remained unchanged within 12 months of treatment. At study entry, RA patients achieving DAS remission had significantly lower IL-6 serum levels than RA patients not achieving this outcome. In particular, having baseline IL-6 serum levels ≤ 8.4 pg/ml, significantly identified naïve to b-DMARDs RA patients more likely to achieve DAS-remission under CTLA4-Ig at 6 months (66.7%) compared to RA patients with baseline IL-6 serum levels > 8.4 pg/ml [15.4%, OR (95%Cis) 11.00 (1.75–55.82)]. Moreover, having CD4^pos^CD25^pos^FoxP3^pos^ cells rate ≥ 6.0% significantly identifies naïve to b-DMARDs early RA patients more likely to achieve DAS remission at 6 months (83.3%) compared to RA patients with baseline CD4^pos^CD25^pos^FoxP3^pos^ cells < 6.0% [16.7%, OR (95% Cis) 25.00 (1.00–336.81)].

**Conclusions:**

Baseline IL-6 serum levels and peripheral blood-derived CD4^pos^ subpopulations are putative novel prognostic biomarkers of CTLA4-Ig response in RA patients.

**Supplementary Information:**

The online version contains supplementary material available at 10.1186/s13075-022-02827-5.

## Background

The introduction of biological disease-modifying drugs (b-DMARDs) has significantly improved the armamentarium of the treatments for rheumatoid arthritis (RA) patients increasing the rate of disease remission achievement after the failure of conventional DMARDs [1]. However, due to disease heterogeneity [2, 3] and b-DMARD distinct modes of actions [1], it will be increasingly necessary to adopt different biomarkers which might support clinical decisions and predict treatment outcome in RA patients. Among the modes of actions directly implicated in the modulation of the inflammation in RA, CTLA4 acts by blocking CD80/86 co-stimulatory receptors on antigen-presenting cells leading to a significant repression of T lymphocyte activation [4]. T-lymphocytes play a crucial role in RA pathogenesis whose aberrant activation phenotype is tightly related to disease activity in RA patients [5]. In particular, a relevant amount of evidence showed that peripheral blood of RA patients is enriched by Th17 lymphocytes compared to healthy controls while controversial data have been produced on peripheral blood Treg rates in RA patients compared to controls [6]. Of biological relevance, synovial tissue of RA patients was found to be enriched of FoxP3^pos^ Tregs consistently with inflammation degree [7–9]. Moreover, CD25^high^ Treg cell rate in synovial tissue-derived cell suspensions of RA patients was found to be significantly higher compared to peripheral blood [10]. Among the different inflammatory soluble factors, interleukin-6 (IL-6), a pleiotropic mediator with a pivotal role in RA pathogenesis, exerts potent immunomodulatory effects on the balance between Th17 cells and CD4^pos^CD25^pos^FoxP3^pos^ regulatory T lymphocytes in RA patients [11].

Based on these issues, the aims of this prospective longitudinal study in RA patients were (i) to assess whether CTLA4-Ig treatment impacts peripheral blood-derived CD4^pos^ cell subpopulation rates, their activation profile, and cytokines milieu in b-DMARD RA patients; (ii) to assess whether baseline, CD4^pos^CD25^pos^FoxP3^pos^, IL6 serum levels together with STAT3 and STAT5 expression might be possible biomarkers of response to CTLA4-Ig in RA patients; and (iii) to test their role as putative prognostic biomarkers of disease remission achievement with CTLA4-Ig therapy within novel personalized medicine approaches for RA.

## Patients and methods

### RA patient enrolment

In this single-center study, 34 patients, fulfilling the 2010 American College of Rheumatology/European League Against Rheumatism (ACR/EULAR) classification criteria for RA [12], were consecutively enrolled between May 2014 and May 2017. RA patients were stratified based on disease duration as follows: 15 RA patients with disease duration less than 3 years (RA cohort 1) and 19 with disease duration more than 3 years [mean ± SEM: 6.8 ± 2.9 years (RA cohort 2)]. At study entry, demographic, clinical, and immunological parameters were recorded. Each enrolled RA patient was naïve to b-DMARDs. After enrollment, each RA patient was treated with CTLA4-Ig according to the current recommendations, and at each study visit, the ACR/EULAR core data set [erythrocyte sedimentation rate (ESR), C-reactive protein (CRP), swollen joint count (SJC), tender joint count (TJC), physician and patient global assessment, pain, and Health Assessment Questionnaire (HAQ)] was recorded. At study entry and at every time point, immunoglobulin A (IgA)- and immunoglobulin M (IgM)-rheumatoid factor (RF) (Orgentec Diagnostika, Bouty, UK) and anti-CCP (ACPA) antibodies (Menarini, Italy) were assessed using commercial enzyme-linked immunosorbent assay (ELISA) or chemiluminescent methods, respectively. The cut-off levels were 20 U/mL for IgM-RF and IgA-RF and 5 U/mL for anti-CCP antibodies. Moreover, each enrolled RA patient was followed within a clinical outpatient setting every 3 months recording the clinical improvement and remission achievement rate based on the DAS and ACR/EULAR criteria [13], respectively. Ten age- and sex-matched healthy controls were enrolled as a comparison group [5 (50.0%) female with a mean ± SEM age of 47.1 ± 9.3 years]. The study protocol was approved by the Università Cattolica del Sacro Cuore Ethical Committee (ID:158, Prot. N. 0031255/17) and all subjects provided signed informed consent to participate in the study.

### Enzyme-linked immunosorbent assay (ELISA) of IL-6, IL-12p70, TGFβ, and IL-10 serum levels

After peripheral blood drawing, each sample was centrifuged at 3500 rpm within 15 min from collection and stored at − 80 °C until analyzed. ELISA kits for the detection of human IL-6, IL-12p70, and TGFβ were obtained from Bio-Techne (UK). The IL-10 detection was assessed with High Sensitivity (HS) ELISA (Bio-Techne, UK). Serum cytokine’s levels were determined according to the manufacturer’s instructions. The IL-6 (sensitivity: 0.7 pg/ml), IL-10 HS (sensitivity: 0.17 pg/ml), IL-12p70 (sensitivity: 5 pg/ml), and TGF-β (sensitivity: 15.4 pg/ml) levels were calculated according to the specific standard curves. TGF-β measurement was performed after its activation (BioTechne, UK). Briefly, latent TGF-β1 serum was activated to its immunoreactive form, using solutions for acid activation and neutralization (Sample Activation Kit 1, R&D Systems®).

### Flow cytometry analysis of CD4^pos^ cell subpopulations

Peripheral blood mononuclear cells (PBMCs) were obtained from peripheral blood of RA patients and healthy controls by Ficoll-Hypaque (Cederlane, Ontario, Canada) density centrifugation (1700 rpm for 45 min at room temperature). PBMCs were resuspended in RPMI media supplement with 100U/ml of penicillin/streptomycin (Corning USA), 2 mM L-glutamine (Corning, USA), and 10% fetal bovine serum (Corning, USA) in aliquots of 2 × 10^6^ cells/ml for flow cytometry analysis.

For the analysis of Th17/Treg cells, the cell suspension was washed in PBS, then the cells were re-suspended in 70 μl of staining buffer (eBioscience, San Diego, CA) and stained at 4 °C for 30 min with mouse anti-human Krome Orange (KO) labeled CD45 (clone J33), Pacific Blue (PB) labeled CD4 (clone 13B8.2), allophycocyanin-750 (APC750) labeled CD25 (clone B 1.49.9) antibodies and with phycoerythrin-Cy7 (PE-Cy7) labeled CD127 (clone R34.34) antibody (Treg-1 cells) (Beckman Coulter, Marseille France). Then, cells were fixed using FOXP3/Transcription Factor Fixation/Permeabilization (eBioscience, San Diego, CA) followed by permeabilization using 1 × permeabilization buffer (eBioscience, San Diego, CA) and stained with mouse anti-human APC-labeled FoxP3 (Treg-2 cells, clone: PCH101) (eBioscience, San Diego, CA), according to the manufacturer’s instruction. Tregs were analyzed according to the commonly used Treg definitions: (1) CD4^pos^CD25^pos^CD127^low^ (Treg1) and (2) CD4^pos^CD25^pos^FoxP3^high^ (Treg2) [14, 15]. For analysis of Th17 cells, PBMCs were washed with PBS and stained with KO-CD45 and PB-CD4 at 4 °C for 30 min. After surface staining, the cells were fixed and permeabilized according to the manufacturer’s instruction and then stained with FITC-IL17-A antibody (Th17 cells, clone eBio64DEC17). All stained cells were acquired on a Navios flow cytometry (Beckman Coulter, Marseille France) and data were analyzed using Kaluza Software (Beckman Coulter, Marseille France). The percentage of Treg1, Treg2, and Th17 cells is given as a percentage within the CD4^pos^ population (Supplementary Fig. 1).

### STAT3 and STAT5 expression in CD4^pos^ cell subset sorted from PB of RA patients

CD4^pos^ cells were isolated from PBMC of the 34 RA patients and 10 healthy controls using CD4 MACS MicroBeads (Miltenyi Biotec, Bergisch Gladbach, Germany) and total RNA was isolated from PB CD4^pos^ cells using the mRNeasy kit (Qiagen). The CD4 cell purity was assessed by FACS analysis (mean ± SD: 97.0 ± 0.7%). The iScript cDNA Synthesis Kit (BioRad Laboratories, Hercules, CA; Qiagen) was used for cDNA preparation following the manufacturer’s instruction. A FastStart Universal Probe Master (04913949001) (Roche Diagnostics, Germany) was used for RT-PCR using the following primers: human signal transducer and activator of transcription (STAT) 3 (STAT3, 100136630), human STAT5 (100136649), and human glyceraldehyde 3-phosphate dehydrogenase (GAPDH, 101128), all for Roche Diagnostics (Germany). Gene expression was evaluated through real-time PCR (Biorad IQ5, Hercules, CA). For the semiquantitative expression of human STAT3 and STAT5 and control GAPDH in CD4^pos^ cells, delta T values were generated after subtraction from the gene of interest (STAT3 or STAT5) Ct value of control (GAPDH) and the relative expression was calculated using the ΔΔCt method (relative gene expression = 2^−(ΔCt test − ΔCt control)^) and is presented in fold increase relative to control.

### Statistical analysis

Statistical analysis was performed using SPSS V. 20.0 (SPSS. Chicago, IL, USA) and Prism software (GraphPad-9, San Diego, CA, USA). Categorical and quantitative variables were described as frequencies, percentage, and mean ± SEM. Data on demographic (RA patients and healthy controls), immunological (RA patients and healthy controls), and clinical features (RA patients) were compared between groups by the non-parametric Mann–Whitney *U* test or *χ*^2^ test, as appropriate. Spearman’s rank correlation test was used for correlation in all analyses. Exploratory univariate data analysis was first conducted to assess adequate event frequency between the outcome and the candidate prognostic factors. A receiver operating characteristic (ROC) curve analysis of IL-6 serum levels and CD4^pos^CD25^pos^FoxP3^pos^ cell rates related to DAS remission achievement after 6 months of CTLA4-Ig treatment were performed to obtain relevant thresholds allowing the prediction of CTLA4-Ig response at baseline. The non-parametric ROC plot uses all the data, makes no parametric assumption, and provides unbiased estimates of sensitivity and specificity. The optimal cut-off point was determined to yield the maximum corresponding sensitivity and specificity. Moreover, a Kaplan–Meier analysis was performed to estimate the probability of occurrence of remission during CTLA4-Ig treatment follow-up among RA patients, and the log-rank test was used to test the differences between subjects divided according to study cohorts and identified cut-off value for IL-6 serum levels at study entry. For all the analyses, a *p* < 0.05 was considered statistically significant and all tests were 2-tailed, unless otherwise indicated.

## Results

### Baseline demographic and clinical characteristics of RA patients treated with CTLA4-Ig

The study included 34 RA patients fulfilling the inclusion criteria of the protocol, who underwent baseline study visit and at least one follow-up visit after entering the study (3 months). During the follow-up period of 12 months, 7 RA patients (20.6% of the general cohort) withdrew from the study, of whom 6 RA patients interrupted the study because of treatment failure (2 after 3 months and 4 after 6 months of CTLA4-Ig treatment) and 1 due to severe infection (testicular abscess) at 6 months of CTLA4-Ig treatment along with persistent RA disease activity. Table 1 summarizes the demographic, immunological, and clinical characteristics of the 34 RA patients enrolled in the study. In particular, RA patients with established disease (cohort 2) were more likely IgM and IgA-RF positive (73.7% and 68.4%, respectively) than early RA (cohort 1) (IgM-RF: 40.0%, *p* = 0.05; IgA-RF: 20.0%, *p* = 0.01) but comparable in terms of ACPA positivity. Moreover, at study entry, RA patients belonging to cohort 2 had more likely bone erosions (73.7%) compared to RA patients of cohort 1 (33.3%, *p* = 0.02). However, disease activity at study entry was comparable between RA patients belonging to RA cohort 1 and RA cohort 2 (Table 1).

**Table 1:** Demographic and clinical characteristics of RA patients treated with CTLA4-Ig at study entry

	Whole RA cohort (*n* = 34)	RA cohort 1 (*n* = 15)	RA cohort 2 (*n* = 19)	p*
Age, years	56.2 ± 14.8	56.5 ± 15.6	55.8 ± 14.5	0.55
Sex, *n*, female (%)	28 (82.4)	14 (93.3)	14 (73.7)	0.14
Disease duration, years	4.3 ± 3.6	1.1 ± 0.9	6.8 ± 2.9	0.001
BMI, Kg/m^2^	25.9 ± 6.7	27.4 ± 8.7	24.7 ± 4.3	0.13
Smoking habit, n (%)	12 (35.5)	4 (26.7)	8 (42.1)	0.35
Anti-CCP^pos^, *n* (%)	28 (82.4)	12 (80.0)	16 (84.2)	0.79
RF-IgM^pos^, *n* (%)	20 (58.8)	6 (40.0)	14 (73.7)	0.05
RF-IgA^pos^, *n* (%)	16 (47.1)	3 (20.0)	13 (68.4)	0.01
ESR, mm/1^^^hour	42.3 ± 23.7	38.7 ± 28.0	45.2 ± 19.3	0.27
CRP, mg/L	16.0 ± 15.6	14.2 ± 14.2	17.5 ± 13.3	0.44
DAS	3.9 ± 0.9	4.0 ± 0.1	3.9 ± 0.9	0.30
CDAI	30.0 ± 10.2	33.9 ± 10.2	27.5 ± 9.7	1.00
SDAI	31.3 ± 10.7	35.2 ± 10.6	28.8 ± 10.3	0.82
HAQ	1.2 ± 0.7	1.3 ± 0.8	1.2 ± 0.7	0.51
Erosive disease, *n* (%)	19 (55.9)	5 (33.3)	14 (73.7)	0.02
csDMARDs (ongoing), *n* (%)	34 (100.0)	15 (100.0)	19 (100.0)	-

Values are mean ± standard deviation unless otherwise indicated. *RA* rheumatoid arthritis, *BMI* body mass index, *ESR* erythrocyte sedimentation rate, *CRP* C-reactive protein, *DAS* Disease Activity Score, *ACPA* anti-citrullinated peptide antibodies, *RF* rheumatoid factor, *CDAI* Clinical Disease Activity Index, *SDAI* Simplified Disease Activity Index, *csDMARDs* conventional synthetic disease-modifying anti-rheumatic drugs, *HAQ* Health Assessment Questionnaire. *Mann–Whitney *U* test or chi-square test as appropriate between RA cohort 1 and RA cohort 2

### CTLA4-Ig treatment induces disease remission in early and established bDMARDs naive RA patients

**Fig. 1:**
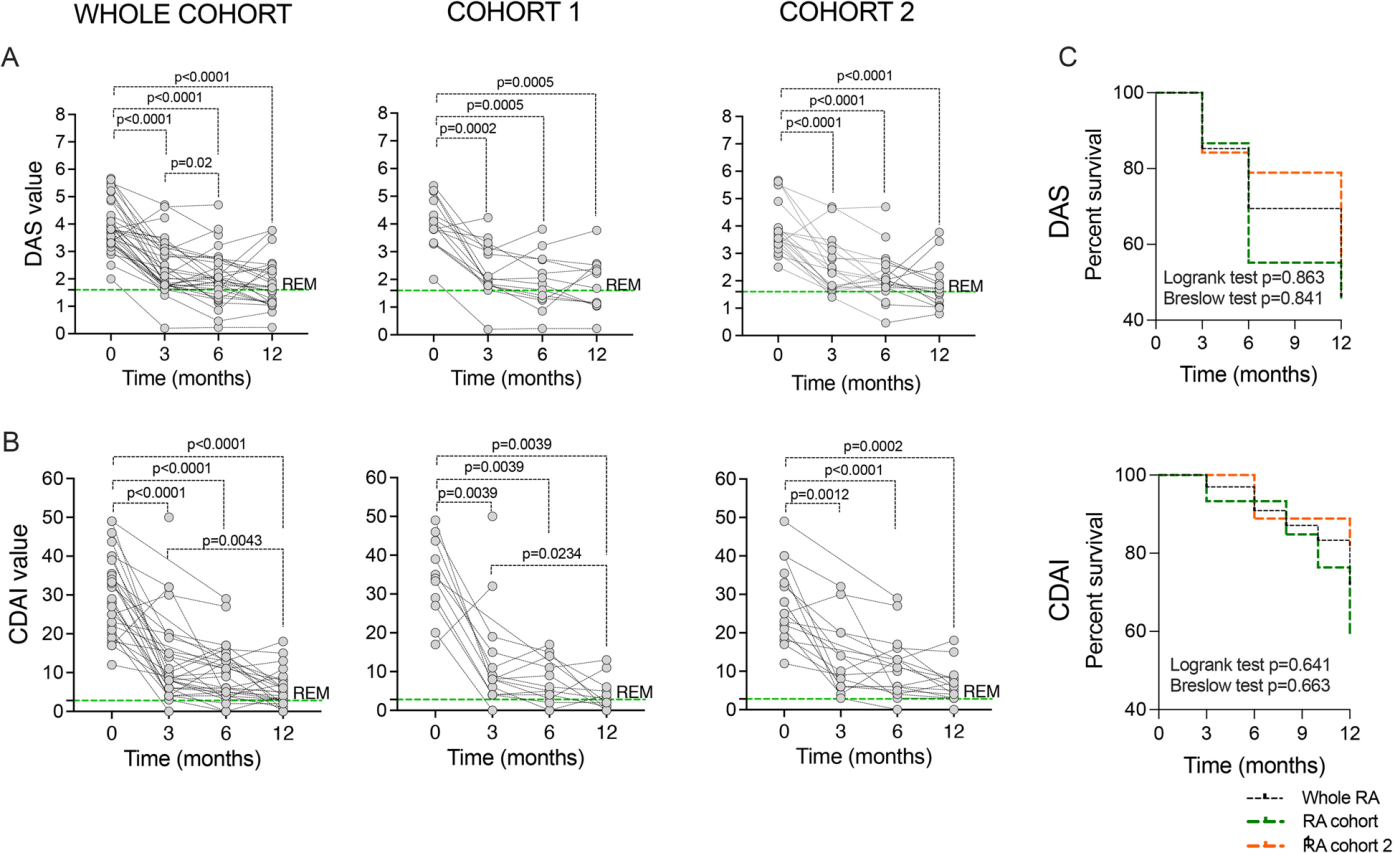
**A**–**C** Clinical outcome of CTLA4-Ig treatment in RA patients.** A** DAS value across 12 months of follow-up of CTLA4-Ig treatment in RA patients (whole cohort, cohort 1, and cohort 2, respectively); *p* values were calculated using Wilcoxon matched-pairs signed rank test comparing DAS at pre-treatment and after 3, 6, and 12 months of CTLA4-Ig administration in RA patients (whole RA cohort, RA cohort 1, or RA cohort 2). **B** CDAI value across 12 months of follow-up of CTLA4-Ig treatment in RA patients (whole cohort, cohort 1, and cohort 2, respectively); *p* values were calculated using Wilcoxon matched-pairs signed rank test comparing CDAI at pre-treatment and after 3, 6, and 12 months of CTLA4-Ig administration in RA patients (whole RA cohort, RA cohort 1, or RA cohort 2). **C** Kaplan–Meier survival curve of treatment with CTLA4-Ig across 12 months of follow-up in RA patients (whole cohort, cohort 1, and cohort 2, respectively) for DAS (log-rank test: chi-square = 0.029 *p* = 0.863; Breslow test: chi-square = 0.346 *p* = 0.841) and CDAI (log-rank test: chi-square = 0.217 *p* = 0.641; Breslow test: chi-square = 0.823 *p* = 0.663). DAS, Disease Activity Score; CDAI, Composite Disease Activity Index; RA, rheumatoid arthritis

The assessed endpoints, DAS-defined and CDAI-defined remission within a 12-month follow-up, were achieved by 16 (47.1%) and 8 (23.5%) RA patients, respectively, without any significant difference between RA cohort 1 and cohort 2 (data not shown). As shown in Fig. 1A, B, disease activity (evaluated by DAS and CDAI) significantly decreased at every time point along CTLA4-Ig treatment compared to study entry in cohorts 1 and 2, respectively, without any significant difference in terms of DAS-defined and CDAI-defined remission achievement in the study cohorts (Fig. 1C). Similar demographic and clinical features at study entry were observed between responders or non-responders RA to CTLA4-Ig treatment in terms of DAS remission (Supplemental Table 1), also stratifying RA patients for disease duration (data not shown). RA achieving CDAI remission after CTLA4-Ig treatment had significantly lower pre-treatment CRP levels (7.6 ± 7.8 mg/L) when compared to RA patients not achieving this clinical outcome (18.6 ± 14.0 mg/L, *p* = 0.03) (Supplemental Table 2).

### CTLA4-Ig treatment significantly reduces IL6 plasma levels and promotes peripheral blood CD4^pos^ cell modulation in RA

**Fig. 2:**
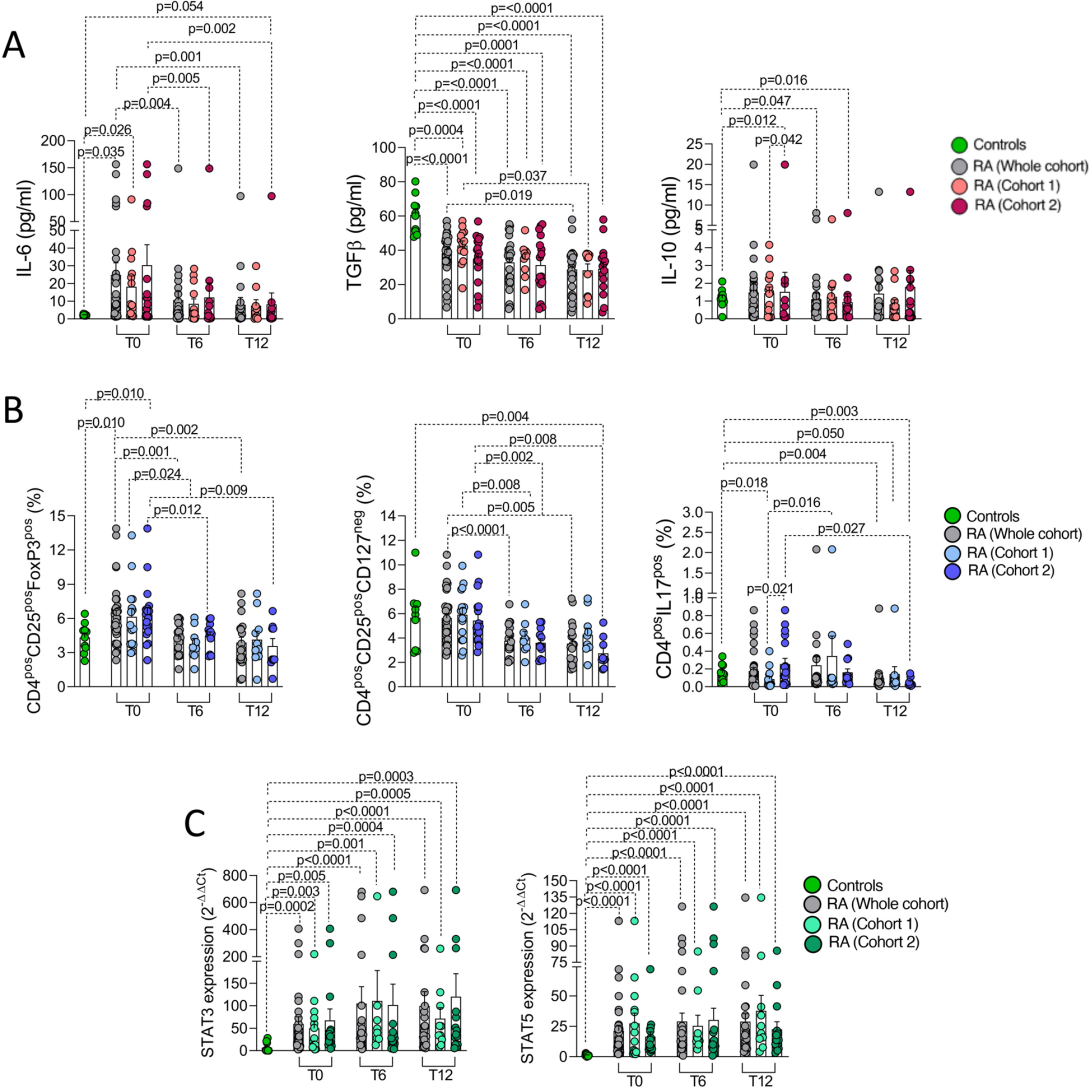
**A**–**C** Modulation of peripheral blood cytokines and T-lymphocyte subpopulations by CTLA4-Ig treatment in RA. **A** IL-6, TGF-b, and IL-10 serum levels of RA patients at study entry and after 6 and 12 months of follow-up of CTLA4-Ig treatment in RA patients (whole cohort, cohort 1, and cohort 2, respectively) and healthy controls. **B** Peripheral blood CD4^pos^CD25^pos^FoxP3^pos^, CD4^pos^CD25^pos^CD127^neg^, and CD4^pos^IL17^pos^ cell percentage at study entry and after 6 and 12 months of follow-up of CTLA4-Ig treatment in RA patients (whole cohort, cohort 1, and cohort 2 respectively) and healthy controls. **C** STAT3 and STAT5 gene expression in CD4^pos^ cells from peripheral blood of RA patients at study entry and after 6 and 12 months of follow-up of CTLA4-Ig treatment (whole cohort, cohort 1, and cohort 2, respectively) and healthy controls. All comparisons between groups were done using the Mann–Whitney *U* test while all comparisons between different time points within the same cohort were done using the Wilcoxon matched-pairs signed rank test. IL, interleukin; TGFβ, transforming growth factor beta; CD, cluster designation; RA, rheumatoid arthritis

To define the effect of CTLA4-Ig treatment on RA patients, IL-6, IL-12p70, IL-10, and TGFβ serum levels were assessed in peripheral blood samples of RA patients. At study entry, RA patients had significantly higher IL-6 (24.9 ± 6.9 pg/ml) and lower TGFβ serum levels (38.3 ± 2.3 pg/ml) than healthy controls (IL-6: 2.2 ± 0.1 pg/ml *p* = 0.035; TGFβ: 60.8 ± 3.4 pg/ml, *p* < 0.0001, respectively) while IL-10 serum levels were comparable (Fig. 2A). The majority of RA patients included in the study showed undetectable IL-12p70 serum levels (< 5 pg/ml) which was not included in the analysis. Moreover, the assessment of longitudinal serum samples of RA patients during CTLA4-Ig treatment showed a significant decrease of IL-6 serum levels from baseline to 6 months (*p* = 0.004) and 12 months of follow-up (*p* = 0.001), respectively, as well as TGFβ serum levels after 12 months of follow-up (*p* = 0.019) (Fig. 2A). Interestingly, at study entry, IL-10 serum levels were higher in RA patients with early disease (IL-10: *p* = 0.042), reaching serum levels comparable to baseline after 6 and 12 months of CTLA4-Ig treatment (Fig. 2A).

Considering peripheral blood T cell phenotype, at study entry, RA patients did not differ from healthy controls in terms of CD4^pos^ cell percentages among CD3^pos^ cells (46.6 ± 4.2% in RA patients and 48.5 ± 2.0% of whole lymphocytes in healthy controls, respectively, *p* = 0.64). However, peripheral blood of RA patients was enriched of CD4^pos^CD25^pos^FoxP3^pos^ cells (6.3 ± 0.5%) than healthy controls (4.3 ± 0.4%, *p* = 0.010) while similar percentages were found considering the CD25/CD127 classification (*p* = 0.95). Moreover, peripheral blood-derived CD4^pos^CD25^pos^FoxP3^pos^ cell rate significantly decreased at 6 (4.2 ± 0.3%, *p* = 0.001) and 12 months (3.9 ± 0.5%, *p* = 0.002) of follow-up of CTLA4-Ig treatment (Fig. 2B). Furthermore, an increased percentage of CD4^pos^IL17^pos^ cells was observed in peripheral blood of RA patients with longer disease duration (cohort 2) when compared to early RA (*p* = 0.021), whose rate significantly decreased after12 months of follow-up of CTLA4-Ig treatment (*p* = 0.027) (Fig. 2B). Peripheral blood-derived CD4^pos^ cells of RA patients were also characterized by a significantly higher STAT3 (59.7 ± 15.9 folds) and STAT5 expression (21.5 ± 4.4 folds) than healthy controls (7.0 ± 3.4 folds; *p* = 0.0002 and 1.3 ± 0.3 folds; *p* < 0.0001, respectively), which conversely remained unchanged during 12 months of CTLA4-Ig treatment (Fig. 2C). Finally, no significant correlations were observed between biological parameters and demographic and clinical features in the RA cohort at enrolment, even considering the RA cohort in a whole or divided based on disease duration (data not shown).

### Baseline features associated with remission achievement at 6 months of CTLA4-Ig treatment in RA patients

**Fig. 3:**
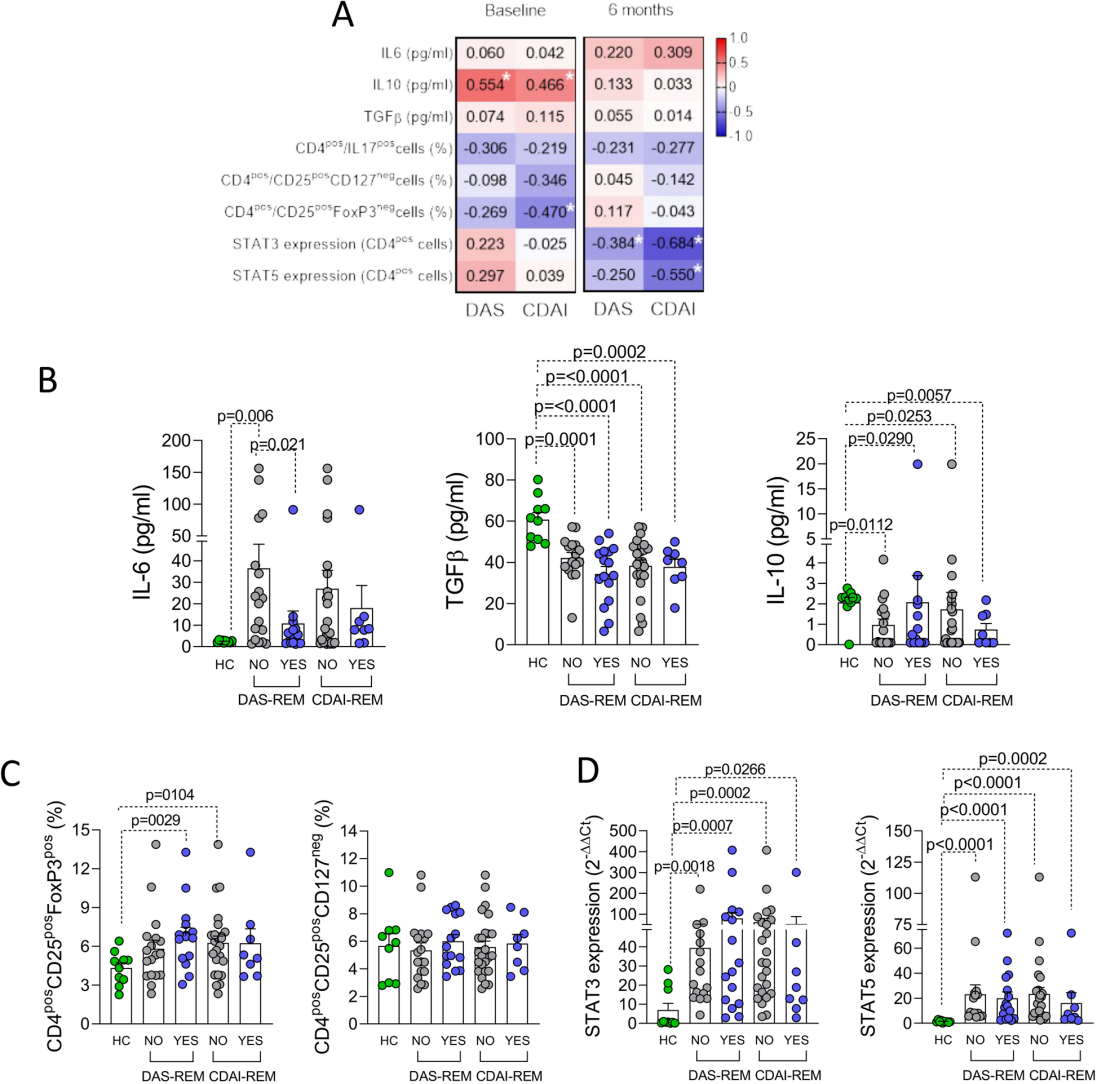
**A**–**D** Baseline inflammatory and peripheral blood CD4^pos^ subpopulations associated with remission achievement under CTLA4-Ig treatment in RA.** A** Spearman correlation test between IL-6, IL-10, TGFβ serum concentrations, CD4^pos^ cell subpopulation rates, STAT3, and STAT5 expression in CD4^pos^ cells in the whole RA cohort with DAS and CDAI at baseline and at 6 months of follow-up of treatment with CTLA4-Ig. Each number indicates the coefficient of the Spearman correlation test. “*” indicates correlations with *p* ≤ 0.05 from the Spearman test. **B** IL-6, TGF-β, and IL-10 serum levels of RA patients at study entry based on the achievement of DAS- and CDAI-defined remission after 6 months of follow-up of CTLA4-Ig treatment in the whole RA cohort and healthy controls. **C** Peripheral blood CD4^pos^CD25^pos^FoxP3^pos^ and CD4^pos^CD25^pos^CD127^neg^ cell percentage at study entry based on the achievement of DAS- and CDAI-defined remission after 6 months of follow-up of CTLA4-Ig treatment in the whole RA cohort and healthy controls. **D** STAT3 and STAT5 expression in peripheral blood-derived CD4^pos^ cells of RA patients at study entry based on the achievement of DAS- and CDAI-defined remission after 6 months of follow-up of CTLA4-Ig treatment in the whole RA cohort and healthy controls. **B**–**D** All comparisons between groups were done using the Mann–Whitney *U* test. HC, healthy controls; IL, interleukin; TGFβ, transforming growth factor beta; CD, cluster designation; RA, rheumatoid arthritis

As shown in Supplementary Table 1, RA patients achieving DAS-defined remission within 12 months of follow-up of CTLA4-Ig treatment did not differ in terms of baseline demographic and clinical parameters when compared to RA patients not reaching the same outcome, with no difference between early and established RA patients (data not shown). However, the assessment of the cytokine milieu and peripheral blood CD4^pos^ cell phenotype in RA patients revealed that disease activity measures (i.e., DAS and CDAI) at baseline were directly related to IL-10 serum levels and inversely with CD4^pos^CD25^pos^FoxP3^pos^ cell rate (Fig. 3A). Moreover, stratifying the whole RA cohort based on the achievement of remission at 6 months of follow-up of CTLA4-Ig treatment, at study entry, RA patients achieving DAS-defined remission had significantly lower IL-6 serum levels (10.8 ± 5.8 pg/ml) compared to RA patients not achieving this outcome (36.6 ± 11.1 pg/ml, *p* = 0.021), while no significant differences were seen in terms of baseline IL-10 or TGFβ serum levels (Fig. 3B). Moreover, despite baseline IL6 serum levels of no-responder RA were significantly higher than responder RA, CTLA4-Ig treatment induced a significant reduction of IL6 serum levels in no-responder patients too, being comparable at 12 months of follow-up (Supplementary Fig. 3A). Considering the peripheral blood CD4^pos^ cell phenotype of the whole RA cohort, there were no significant differences in terms of pre-treatment peripheral blood CD4^pos^CD25^pos^FoxP3^pos^ cell rate. However, a significant decrease of CD4^pos^CD25^pos^FoxP3^pos^ cell rate was found within 12 months of CTLA4-Ig treatment despite treatment response (Supplementary Fig. 3B). Moreover, in the whole RA cohort, there were no significant differences in terms of pre-treatment STAT3 and STAT5 expression in peripheral blood-derived CD4^pos^ cells based on the achievement of DAS- and CDAI-defined remission at 6 months of treatment with CTLA4-Ig (Fig. 3C, D). Moreover, correlation analysis revealed that, after 6 months of CTLA4-Ig treatment, disease activity measures (i.e., DAS and CDAI, respectively) were inversely related to STAT3 and STAT5 expression in peripheral blood CD4^pos^ cells (Fig. 3A).

**Fig. 4:**
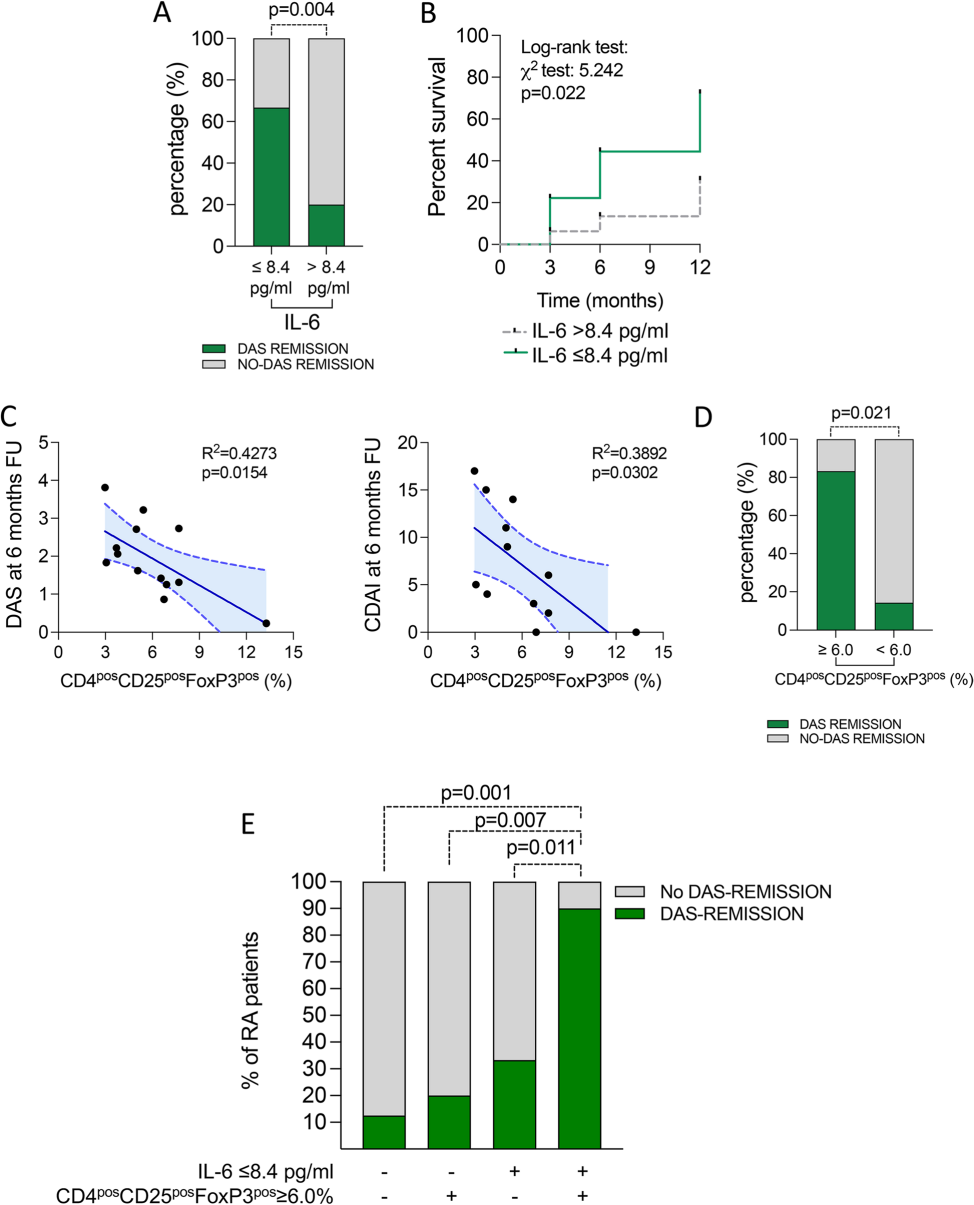
**A**–**E** Baseline inflammatory and peripheral blood CD4^pos^ subpopulations associated with remission achievement under CTLA4-Ig treatment in b-DMARDs naive RA. **A** Rate of naive to b-DMARDs RA achieving DAS remission at 6 months of treatment with CTLA4-Ig based on pre-treatment IL-6 serum levels. **B** Kaplan–Meier survival curve of naive to b-DMARDs RA achieving DAS remission at 6 months of treatment with CTLA4-Ig based on pre-treatment IL-6 serum levels. **C** Spearman correlation test between composite disease activity indices (i.e., DAS and CDAI, respectively) and peripheral blood CD4^pos^CD25^pos^FoxP3^pos^ cell rate in early naive to b-DMARDs RA at 6 months of treatment with CTLA4-Ig. **D** Rate of early naive to b-DMARDs RA achieving DAS remission at 6 months of treatment with CTLA4-Ig based on pre-treatment CD4^pos^CD25^pos^FoxP3^pos^ cells rate; *p* value was calculated using the chi-square test. **E** Rate of naive to b-DMARDs RA achieving DAS remission at 6 months of treatment with CTLA4-Ig based on pre-treatment IL-6 serum levels and/or CD4^pos^CD25^pos^FoxP3^pos^ cell rate. *p* values were calculated using the chi-square test; RA, rheumatoid arthritis; DAS, Disease Activity Score; CDAI, Composite Disease Activity Index; CD, cluster designation; b-DMARDs, biologic disease-modifying anti-rheumatic drugs

Moreover, in the assessment of the possible predictive power of putative biomarkers of treatment response to CTLA4-Ig in the whole RA cohort, ROC curve analysis identified baseline IL-6 serum levels significantly discriminating, at pre-treatment stage, RA patients achieving DAS remission within 6 months. In particular, having baseline IL-6 serum levels ≤ 8.4 pg/ml [(AUC: 0.735 ± 0.089) *p* = 0.02] (Supplementary Fig. 2A), significantly identified naïve to bDMARDs RA patients more likely to achieve DAS-defined remission under CTLA4-Ig treatment at 6 months (66.7%) compared to RA patients with baseline IL-6 serum levels > 8.4 pg/ml [15.4%, *p* = 0.005 (*χ*^2^ test), OR (95%Cis): 11.00 (1.75–55.82)] (Fig. 4A). Moreover, RA patients with lower pre-treatment IL-6 serum levels reached more likely DAS remission within 12 months of CTLA4-Ig treatment (log-rank test: *p* = 0.022) (Fig. 4B).

When considering RA cohort 1 only, peripheral blood CD4^pos^CD25^pos^FoxP3^pos^ cells rate at baseline inversely correlated with disease activity (i.e., DAS and CDAI) at 6 months FU of CTLA4-Ig (Fig. 4C). Moreover, ROC curve analysis revealed that, at baseline, having CD4^pos^CD25^pos^FoxP3^pos^ cell rate ≥ 6.0% [(AUC) 95% CI: 0.66 (0.57–0.74), *p* = 0.001] (Supplementary Fig. 2) significantly identifies naïve to bDMARDs early RA patients more likely to achieve DAS remission under CTLA4-Ig treatment at 6 months (83.3%) compared to RA patients with baseline CD4^pos^CD25^pos^FoxP3^pos^ cells < 6.0% [16.7%, *p* = 0.021 (*χ*^2^ test), OR (95% Cis): 25.00 (1.00 – 336.81)] (Fig. 4D).

Finally, considering the whole cohort receiving CTLA4-Ig, RA patients having pre-treatment IL-6 serum levels ≤ 8.4 pg/ml and CD4^pos^CD25^pos^FoxP3^pos^ cell rate ≥ 6.0% had the highest rate of DAS-remission at 6 months (90.0%) compared to RA patients having just one (IL-6 serum levels ≤ 8.4: 33.3%, *p* = 0.011 or CD4^pos^CD25^pos^FoxP3^pos^ cell rate ≥ 6.0%: 20.0%, *p* = 0.007) or none of these features (12.5%, *p* = 0.001) (Fig. 4E). Moreover, no significant differences were observed in STAT3 and STAT5 expression in peripheral blood-derived CD4^pos^ cells in RA patients treated with CTLA4-Ig based on the fulfillment of the cut-off values of IL6 plasma levels and CD4^pos^CD25^pos^FoxP3^pos^ cell rates associated with DAS remission achievement at 6 months FU (Supplementary Fig. 3).

## Discussion

This prospective longitudinal study investigated the impact of CTLA4-Ig treatment on peripheral blood-derived CD4^pos^ cells in RA patients in terms of cell phenotype and cytokine milieu involved in the unbalanced inflammatory cascade of the disease and their potential role as a prognostic biomarker of treatment response. In particular, we addressed how CTLA-4Ig might impact peripheral blood-derived CD4^pos^ cell subsets (Tregs and Th17), in combination with the modulation of IL6, IL-10, and TGFβ in naïve to bDMARDs RA. We found that CTLA4-Ig significantly modulates blood-derived CD4^pos^ cell phenotype and represses soluble inflammatory molecules linked to successful treatment response in active RA patients.

It is well established that CTLA4-Ig exerts its therapeutic effects in RA patients, acting on CD80/CD86 molecules on the cellular surface of antigen-presenting cells (APC) by blocking the second signal as well as licensing APC to express IDO [16, 17]. In RA patients, the immunological polarization of T-lymphocytes might be triggered by an unbalanced cytokine milieu characterizing RA inflammation [18–20]. In particular, IL-6 is a pleiotropic mediator with a pivotal role in RA pathogenesis [21], exerting potent immunomodulatory effects on the balance between Th17 cells and CD4^pos^CD25^pos^FoxP3^pos^ regulatory T lymphocytes in RA patients [11]. Moreover, among cytokines with immune-modulatory properties, IL12p70 was found to promote proliferation of IL2-dependent T cells, enhancing the expression of CD25 on CD4^pos^ Th1 cells [22], as well as to control autoimmune inflammation, as demonstrated in IL12p35KO mice [23]. These data support that different soluble markers might be associated with peripheral blood-derived CD4^pos^ cell polarization, suggesting their putative role as biomarkers of treatment response to CTLA4-Ig in RA patients. Along this line, CTLA4-Ig treatment was found to significantly reduce T-cell repertoire restriction [24] and to significantly increase peripheral blood-derived CD4^pos^CD25^pos^FoxP3^pos^ regulatory T lymphocyte rate in RA patients together with their apoptosis [25]. However, conflicting data were produced about the relation between peripheral blood-derived activated Th17 cells and CTLA4-Ig induced disease remission in RA patients [26, 27], while peripheral blood-derived activated Treg cells before treatment were found to be significantly higher in RA patients achieving disease remission with CTLA4-Ig [26]. In our study, we found that CTLA4-Ig treatment significantly reduces the rates of peripheral blood-derived CD4^pos^CD25^pos^FoxP3^pos^ cells in RA patients regardless of their disease duration. Moreover, considering peripheral blood-derived CD4^pos^IL17^pos^ cells, RA patients with disease duration longer than 3 years have significantly higher percentages compared to early RA patients, requiring at least 12 months of CTLA4-Ig treatment to significantly reduce their rates, suggesting a distinct dynamism of CTLA4-Ig induced modulation on CD4^pos^ cell phenotype. Concomitantly, a significant reduction of IL-6 serum levels was detected under CTLA4-Ig treatment in RA patients regardless of their disease duration.

In RA patients, TGFβ exerts a profound control on the adaptive immune response [28], controlling the maturation and function of a specialized subset (i.e., CD4^pos^Tregs) which are of fundamental importance for auto-reactivity control [29]. Moreover, IL6 acts via the JAK/STAT pathways, namely STAT3 and STAT5 that are two transcription factors known to control the differentiation of Th17 and Treg cells, respectively [30]. In our study, peripheral blood-derived CD4^pos^ cells from RA patients were enriched of STAT3 and STAT5 compared to CD4^pos^ cells isolated from healthy controls, despite their activation status, i.e., phosphorylation was not determined. Moreover, despite CTLA4-Ig treatment did not impact STAT3 and STAT5 expression in peripheral blood-derived CD4^pos^ cells in RA patients, their STAT3 and STAT5 expression was inversely correlated with CDAI value after 6 months of CTLA4-Ig treatment.

To date, limited biomarkers of CTLA4-Ig treatment prognosis are available in the clinic. Among them, seropositivity for ACPA autoantibodies, at their highest titers, arose as a putative biomarker of a successful clinical treatment response in RA patients [31]. Recently, a study comparing the impact of different biological DMARDs (namely tocilizumab, certolizumab pegol, and CTLA4-Ig) showed that different b-DMARDs have distinct effects on CD4^pos^ cell phenotype in RA patients. In particular, CTLA4-Ig treatment significantly represses all subtypes of peripheral blood-derived Tregs in early RA patients differently from IL-6R and TNF-β inhibitors whose effect was limited to CTLA4^pos^ cells, which represent activated Tregs directly related to the disease burden [32]. In our study, stratifying the whole RA cohort based on the achievement of DAS remission at 6 months under CTLA4-Ig treatment, we found that RA patients achieving this clinical outcome had, at baseline of CTLA4-Ig therapy, significantly lower serum levels of IL6 compared to RA patients not achieving the same clinical outcome, identifying a cut-off value of 8.4 pg/ml for IL-6 serum levels characterizing RA patients with the lowest chance of DAS remission achievement at 6 months of CTLA4-Ig therapy, regardless of disease duration. In addition, considering the pre-treatment CD4^pos^ cell phenotype, early RA patients with pre-treatment enrichment of CD4^pos^CD25^pos^FoxP3^pos^ rates (≥ 6.0%) among peripheral blood-derived CD4^pos^ cells, had the highest chance of DAS remission achievement at 6 months of CTLA4-Ig treatment. Interestingly, RA patients with IL-6 serum levels < 8.4 pg/ml and CD4^pos^CD25^pos^FoxP3^pos^ rates ≥ 6.0% had the highest chance of DAS remission achievement under CTLA4-Ig treatment.

In conclusion, CTLA4-Ig enables the modulation of peripheral blood-derived CD4^pos^ cell subtypes in RA patients in terms of Th17 CD4^pos^ cells decrease and a trend toward the normalization of Tregs in parallel with an effect on IL-6 burden. Moreover, pre-treatment IL-6 serum levels and CD4^pos^CD25^pos^FoxP3^pos^ rates arose as putative biomarkers of successful treatment response to CTLA4-Ig in RA patients. Prospective studies dissecting the therapeutic effects of CTLA4-Ig on the synovial compartment are needed to confirm the dynamism of CD4^pos^ cell subpopulations and might give novel insights on our data in naïve and established RA patients showing that CTLA-4Ig promotes an immunological re-setting of the deranged immunological balance with particular focus on IL-6 levels and T-Regs that arose as the strongest biomarkers of the clinical outcome. These biological data may clearly offer an explanation and support for the results of the NORD-STAR trial [33].

## Supplementary Information

**Additional file 1: Supplementary Table 1**. Demographic and clinical characteristics of RA responders to CTLA4-Ig at study entry compared to no-responders (DAS-remission). Values are mean ± standard deviation unless otherwise indicated. RA: Rheumatoid Arthritis; BMI: body mass index; ESR: erythrocyte sedimentation rate; CRP: C-reactive protein; DAS: disease activity score; ACPA: anti-citrullinated peptide antibodies; RF: rheumatoid factor; CDAI: clinical disease activity index; SDAI: simplified disease activity index; csDMARDs: conventional synthetic Disease Modifying Anti-Rheumatic Drugs; HAQ: Health Assessment Questionnaire. *Mann–Whitney U-test or Chi-square test as appropriate between RA Cohort 1 and RA Cohort 2. **Supplementary Table 2.** Demographic and clinical characteristics of RA responders to CTLA4-Ig at study entry compared to no-responders (CDAI-remission). Values are mean ± standard deviation unless otherwise indicated. RA: Rheumatoid Arthritis; BMI: body mass index; ESR: erythrocyte sedimentation rate; CRP: C-reactive protein; DAS: disease activity score; ACPA: anti-citrullinated peptide antibodies; RF: rheumatoid factor; CDAI: clinical disease activity index; SDAI: simplified disease activity index; csDMARDs: conventional synthetic Disease Modifying Anti-Rheumatic Drugs; HAQ: Health Assessment Questionnaire. *Mann–Whitney U-test or Chi-square test as appropriate between RA Cohort 1 and RA Cohort 2.

**Additional file 2: Supplementary Fig. 1.** Flow cytometry gating scheme to identify Th17/Treg subpopulations in human CD4pos cells from peripheral blood mononuclear cells of RA patients. Single-cell suspension from RA patients were stained with a combination of 6 antibodies as described in Materials and methods. After gating the singlets, lymphocytes were identified based on their side-scatter properties and expression of CD45. CD4 was used to identify CD4^pos^ T cells among the previously selected lymphocytes. Subsequently, Tregs were analyzed on CD4 according to the commonly used Treg definitions: CD4^pos^CD25^pos^CD127^low^ (Treg1) and CD4^pos^CD25^pos^FoxP3^high^ (Treg2). Moreover, IL17-A expression was measured on CD4^pos^ cells (Th17). All stained cells were acquired on a Navios flow-cytometer (Beckman Coulter, Marseille France) and data were analysed using Kaluza Software (Beckman Coulter, Marseille France). Percentage of Treg1, Treg2 and Th17 cells is given as percentage within the CD4^pos^ population.

**Additional file 3: Supplementary Fig. 2.** ROC curves for IL-6 **(A)** and CD4^pos^CD25^pos^FoxP3^pos^ cells rate **(B)** significantly distinguishing RA patients achieving DAS-defined remission at 6 months of CTLA4-Ig treatment.

**Additional file 4: Supplementary Fig. 3**. IL-6 serum levels (A) and peripheral blood CD4^pos^CD25^pos^FoxP3^high^ subset distribution (B) across 12 months follow-up in RA patients treated with CTLA4-Ig based on the achievement of DAS-remission.

**Additional file 5: Supplementary Fig. 4.** STAT3 and STAT5 expression in CD4^pos^ cells from peripheral blood of RA patients treated with CTLA4-Ig based on the fulfillment of cut-off values of IL6 plasma levels and CD4^pos^CD25^pos^FoxP3^pos^ rates associated with DAS-remission achievement at 6 months.

## Data Availability

The datasets used and/or analyzed during the current study are available from the corresponding authors on reasonable request.
