# Correction: Peripheral blood CD4^pos^CD25^pos^FoxP3^pos^ cells and inflammatory cytokines as biomarkers of response in rheumatoid arthritis patients treated with CTLA4-Ig

**DOI:** 10.1186/s13075-022-02866-y

**Published:** 2022-07-25

**Authors:** Elisa Gremese, Barbara Tolusso, Luca Petricca, Clara Di Mario, Maria Rita Gigante, Gianfranco Ferraccioli, Stefano Alivernini

**Affiliations:** 1 Division of Clinical Immunology, Catholic University of the Sacred Heart, Fondazione Policlinico Universitario A. Gemelli IRCCS, Via Giuseppe Moscati 31, 00168 Rome, Italy; 2 Immunology Core Facility, Gemelli Science Technological Park, GSTeP, Fondazione Policlinico Universitario A. Gemelli IRCCS, Rome, Italy; 3 Università Cattolica del Sacro Cuore, Rome, Italy; 4 Division of Rheumatology, Catholic University of the Sacred Heart, Fondazione Policlinico Universitario A. Gemelli IRCCS, Largo Agostino Gemelli 1, 00168 Rome, Italy

**Correction: Arthritis Res Ther 24, 143 (2022)**

**https://doi.org/10.1186/s13075-022-02827-5**

Following publication of the original article [1], the authors identified an error in the author name of Elisa Gremese. The given name and family name were erroneously transposed.

The incorrect author name is: Gremese Elisa

The correct author name is: Elisa Gremese

The author group has been updated above and the original article [1] has been corrected.
