# Supplementary material for: Peripheral blood CD4posCD25posFoxP3pos cells and inflammatory cytokines as biomarkers of response in rheumatoid arthritis patients treated with CTLA4-Ig
Source: Arthritis Res Ther. 2022 Jun 15;24:143. doi: 10.1186/s13075-022-02827-5 (PMC9199213; doi:10.1186/s13075-022-02827-5)
Supplement: Supplementary file 1 — Additional file 1: Supplementary Table 1. Demographic and clinical characteristics of RA responders to CTLA4-Ig at study entry compared to no-responders (DAS-remission). Values are mean ± standard deviation unless otherwise indicated. RA: Rheumatoid Arthritis; BMI: body mass index; ESR: erythrocyte sedimentation rate; CRP: C-reactive protein; DAS: disease activity score; ACPA: anti-citrullinated peptide antibodies; RF: rheumatoid factor; CDAI: clinical disease activity index; SDAI: simplified disease activity index; csDMARDs: conventional synthetic Disease Modifying Anti-Rheumatic Drugs; HAQ: Health Assessment Questionnaire. *Mann–Whitney U-test or Chi-square test as appropriate between RA Cohort 1 and RA Cohort 2. Supplementary Table 2. Demographic and clinical characteristics of RA responders to CTLA4-Ig at study entry compared to no-responders (CDAI-remission). Values are mean ± standard deviation unless otherwise indicated. RA: Rheumatoid Arthritis; BMI: body mass index; ESR: erythrocyte sedimentation rate; CRP: C-reactive protein; DAS: disease activity score; ACPA: anti-citrullinated peptide antibodies; RF: rheumatoid factor; CDAI: clinical disease activity index; SDAI: simplified disease activity index; csDMARDs: conventional synthetic Disease Modifying Anti-Rheumatic Drugs; HAQ: Health Assessment Questionnaire. *Mann–Whitney U-test or Chi-square test as appropriate between RA Cohort 1 and RA Cohort 2. [file 13075_2022_2827_MOESM1_ESM.docx]

**SUPPLEMENTARY TABLES**

**Supplementary Table 1. Demographic and clinical characteristics of RA responders to CTLA4-Ig at study entry compared to no-responders (DAS-remission).**

|  | Whole RA cohort | |  |
| --- | --- | --- | --- |
|  | DAS remission  (n=16) | No DAS-Remission  (n=18) | P* |
| Age, years | 54.2 ± 16.6 | 57.9 ± 13.1 | 0.55 |
| Sex, n. female (%) | 12 (75.0) | 16 (88.9) | 0.29 |
| Disease duration, years | 5.3 ± 4.4 | 3.4 ± 2.6 | 0.24 |
| BMI, Kg/m^2^ | 24.1 ± 7.1 | 27.5 ± 6.1 | 0.13 |
| Smoking habit, n(%) | 5 (31.2) | 7 (38.9) | 0.64 |
| Anti-CCP^pos,^ n(%) | 14 (87.5) | 14 (77.8) | 0.46 |
| RF-IgM^pos,^ n(%) | 9 (56.2) | 11 (61.1) | 0.77 |
| RF-IgA^pos,^ n(%) | 9 (56.2) | 7 (38.9) | 0.31 |
| ESR, mm/1^^^hour | 37.8 ± 22.5 | 46.4 ± 24.0 | 0.27 |
| CRP, mg/L | 13.8 ± 12.1 | 18.0 ± 14.8 | 0.44 |
| DAS | 3.8 ± 1.0 | 4.0 ± 0.8 | 0.30 |
| CDAI | 29.8 ± 9.5 | 30.2 ± 11.0 | 1.00 |
| SDAI | 30.6 ± 9.7 | 31.8 ± 11.6 | 0.82 |
| HAQ | 1.1 ± 0.8 | 1.3 ± 0.7 | 0.51 |
| Erosive disease, n(%) | 9 (56.2) | 10 (55.6) | 0.97 |
| csDMARDs (ongoing), n (%) | 16 (100.0) | 18 (100.0) | - |

Values are mean ± standard deviation unless otherwise indicated. RA: Rheumatoid Arthritis; BMI: body mass index; ESR: erythrocyte sedimentation rate; CRP: C-reactive protein; DAS: disease activity score; ACPA: anti-citrullinated peptide antibodies; RF: rheumatoid factor; CDAI: clinical disease activity index; SDAI: simplified disease activity index; csDMARDs: conventional synthetic Disease Modifying Anti-Rheumatic Drugs; HAQ: Health Assessment Questionnaire. *Mann-Whitney U-test or Chi-square test as appropriate between RA Cohort 1 and RA Cohort 2.

**Supplementary Table 2: Demographic and clinical characteristics of RA responders to CTLA4-Ig at study entry compared to no-responders (CDAI-remission).**

|  | Whole RA cohort | |  |
| --- | --- | --- | --- |
|  | CDAI remission  (n=8) | No CDAI-Remission  (n=26) | P* |
| Age, years | 50.9 ± 21.2 | 57.8 ± 12.3 | 0.51 |
| Sex, n. female (%) | 8 (100) | 20 (76.9) | 0.13 |
| Disease duration, years | 3.5 ± 4.0 | 4.5 ± 3.6 | 0.48 |
| BMI, Kg/m^2^ | 23.0 ± 5.7 | 26.8 ± 6.8 | 0.18 |
| Smoking habit, n(%) | 3 (37.5) | 9 (34.6) | 0.88 |
| Anti-CCP^pos,^ n(%) | 6 (75.0) | 22 (84.6) | 0.53 |
| RF-IgM^pos,^ n(%) | 4 (50.0) | 16 (61.5) | 0.56 |
| RF-IgA^pos,^ n(%) | 3 (37.5) | 13 (50.0) | 0.54 |
| ESR, mm/1^^^hour | 36.6 ± 24.1 | 44.1 ± 23.3 | 0.53 |
| CRP, mg/L | 7.6 ± 7.8 | 18.6 ± 14.0 | 0.03 |
| DAS | 3.6 ± 1.0 | 4.0 ± 0.9 | 0.39 |
| CDAI | 31.1 ± 8.9 | 29.7 ± 10.9 | 0.64 |
| SDAI | 32.2 ± 8.9 | 31.0 ± 11.5 | 0.72 |
| HAQ | 0.8 ± 0.5 | 1.3 ± 0.8 | 0.15 |
| Erosive disease, n(%) | 3 (37.5) | 16 (61.5) | 0.23 |
| csDMARDs (ongoing), n (%) | 16 (100.0) | 18 (100.0) | - |

Values are mean ± standard deviation unless otherwise indicated. RA: Rheumatoid Arthritis; BMI: body mass index; ESR: erythrocyte sedimentation rate; CRP: C-reactive protein; DAS: disease activity score; ACPA: anti-citrullinated peptide antibodies; RF: rheumatoid factor; CDAI: clinical disease activity index; SDAI: simplified disease activity index; csDMARDs: conventional synthetic Disease Modifying Anti-Rheumatic Drugs; HAQ: Health Assessment Questionnaire. *Mann-Whitney U-test or Chi-square test as appropriate between RA Cohort 1 and RA Cohort 2.
