# Supplementary figures and images for: Peripheral blood CD4posCD25posFoxP3pos cells and inflammatory cytokines as biomarkers of response in rheumatoid arthritis patients treated with CTLA4-Ig
Source: Arthritis Res Ther. 2022 Jun 15;24:143. doi: 10.1186/s13075-022-02827-5 (PMC9199213; doi:10.1186/s13075-022-02827-5)

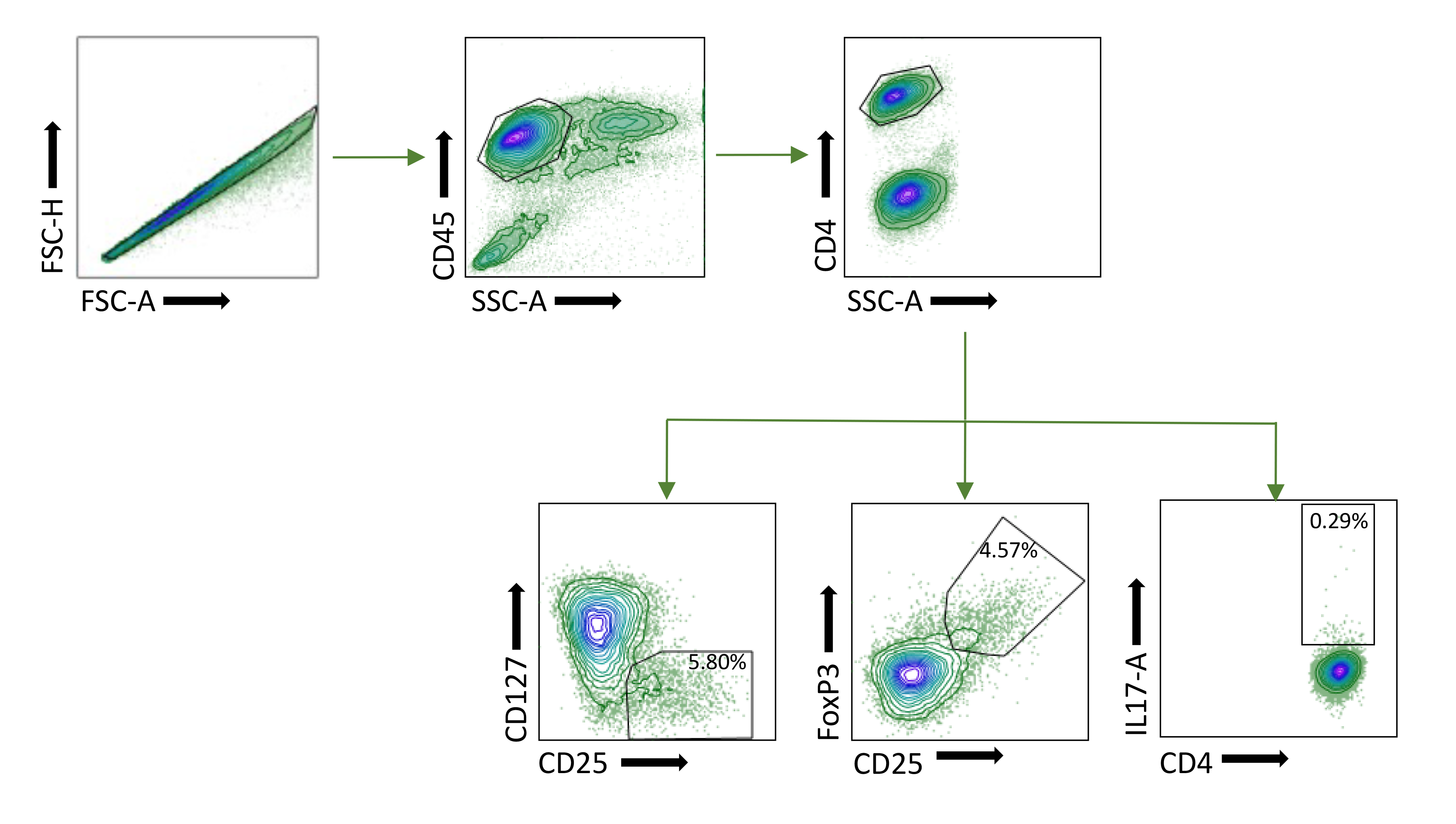

Supplement: Supplementary file 2 — Additional file 2: Supplementary Fig. 1. Flow cytometry gating scheme to identify Th17/Treg subpopulations in human CD4pos cells from peripheral blood mononuclear cells of RA patients. Single-cell suspension from RA patients were stained with a combination of 6 antibodies as described in Materials and methods. After gating the singlets, lymphocytes were identified based on their side-scatter properties and expression of CD45. CD4 was used to identify CD4pos T cells among the previously selected lymphocytes. Subsequently, Tregs were analyzed on CD4 according to the commonly used Treg definitions: CD4posCD25posCD127low (Treg1) and CD4posCD25posFoxP3high (Treg2). Moreover, IL17-A expression was measured on CD4pos cells (Th17). All stained cells were acquired on a Navios flow-cytometer (Beckman Coulter, Marseille France) and data were analysed using Kaluza Software (Beckman Coulter, Marseille France). Percentage of Treg1, Treg2 and Th17 cells is given as percentage within the CD4pos population. [file 13075_2022_2827_MOESM2_ESM.tif]

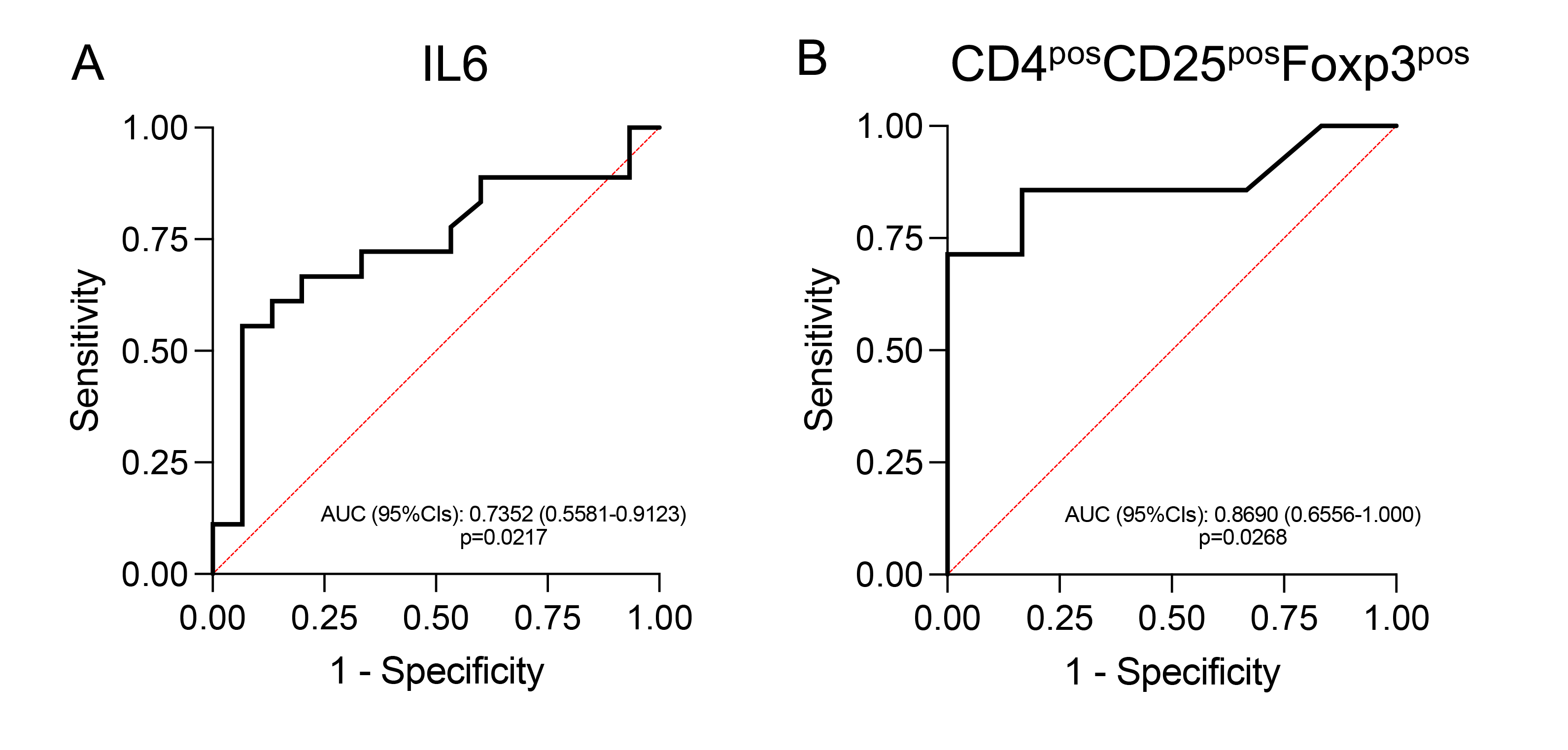

Supplement: Supplementary file 3 — Additional file 3: Supplementary Fig. 2. ROC curves for IL-6 (A) and CD4posCD25posFoxP3pos cells rate (B) significantly distinguishing RA patients achieving DAS-defined remission at 6 months of CTLA4-Ig treatment. [file 13075_2022_2827_MOESM3_ESM.tif]

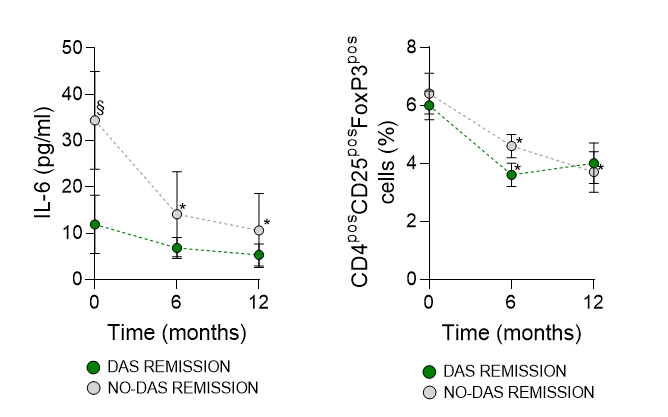

Supplement: Supplementary file 4 — Additional file 4: Supplementary Fig. 3. IL-6 serum levels (A) and peripheral blood CD4posCD25posFoxP3high subset distribution (B) across 12 months follow-up in RA patients treated with CTLA4-Ig based on the achievement of DAS-remission. [file 13075_2022_2827_MOESM4_ESM.tif]

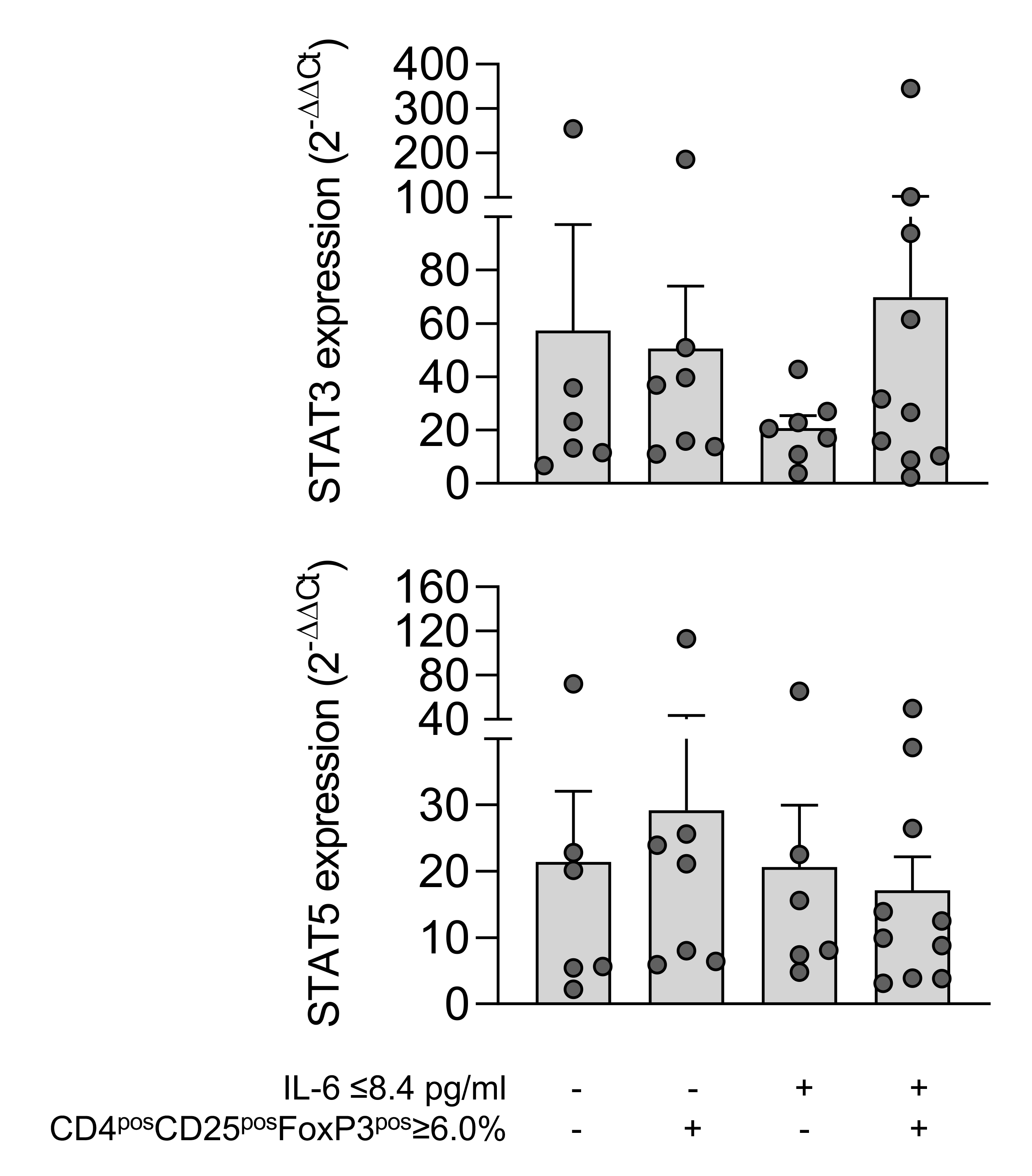

Supplement: Supplementary file 5 — Additional file 5: Supplementary Fig. 4. STAT3 and STAT5 expression in CD4pos cells from peripheral blood of RA patients treated with CTLA4-Ig based on the fulfillment of cut-off values of IL6 plasma levels and CD4posCD25posFoxP3pos rates associated with DAS-remission achievement at 6 months. [file 13075_2022_2827_MOESM5_ESM.tif]
